# Efficacy and safety of the novel GlyT1 inhibitor BI 425809 in Alzheimer’s dementia: a randomized controlled trial

**DOI:** 10.1186/s13195-023-01163-3

**Published:** 2023-01-28

**Authors:** Glen Wunderlich, Zuzana Blahova, Miguel Garcia, Frank Jessen

**Affiliations:** 1 Boehringer Ingelheim Pharmaceuticals Inc., 900 Ridgebury Road, Ridgefield, CT 06877 USA; 2 Boehringer Ingelheim RCV GmbH & Co. KG, Vienna, Austria; 3 Department of Psychiatry, Medical Faculty, University Hospital Cologne, 50924 Cologne, Germany; 4 German Center for Neurodegenerative Diseases (DZNE), Bonn/Cologne, Germany; 5 Excellence Cluster on Cellular Stress Responses in Aging-Associated Diseases (CECAD), Medical Faculty, University of Cologne, Cologne, Germany

**Keywords:** Alzheimer’s disease, Alzheimer’s dementia, Phase II, BI 425809, Glycine transporter-1 inhibitor, Randomized controlled trial, Proof-of-concept

## Abstract

**Background:**

This phase II proof-of-concept study assessed the efficacy and safety of BI 425809, a novel selective glycine transporter-1 inhibitor, for the treatment of cognitive impairment associated with probable Alzheimer’s disease dementia.

**Methods:**

This 12-week, multicenter, double-blind, placebo-controlled, parallel-group study randomized (1:1:1:1:1) patients with mild-to-moderate probable Alzheimer’s disease dementia to BI 425809 2, 5, 10, and 25 mg or placebo once daily. The primary efficacy endpoint was the change from baseline in Alzheimer’s Disease Assessment Scale-Cognitive Subscale 11-item total score after 12 weeks of treatment. Safety was also assessed.

**Results:**

Six hundred and ten male and female patients were randomized to BI 425809 2 mg (*n* = 123), 5 mg (*n* = 122), 10 mg (*n* = 122), and 25 mg (*n* = 123) or placebo (*n* = 120). Approximately 47% (*n* = 286) were male; the mean (standard deviation) age was 72.9 (7.7) years. Treatment compliance was above 97% for all dose groups. The Mini-Mental State Examination category on the median score was < 22 in 47% (*n* = 287) of patients and ≥ 22 in 53% (*n* = 322) of patients. No significant, non-flat dose–response relationship was detected for the primary endpoint (adjusted *p*-value > 0.76 for all models). BI 425809 was generally well-tolerated. Overall, 47.9% (*n* = 292) of patients reported at least one adverse event during the trial; the frequency of patients with investigator-defined drug-related adverse events was similar in all treatment groups, ranging from 15.4 to 19.5% across the BI 425809 treatment groups and 15.8% for placebo.

**Conclusions:**

No clinically meaningful changes from baseline were observed following treatment with BI 425809 in patients with mild-to-moderate probable Alzheimer’s disease dementia.

**Trial registration:**

ClinicalTrials.gov NCT02788513 (1346-0023). Registered on June 2, 2016. EU Clinical Trials Register 2015-005438-24. Registered on May 6, 2016

**Supplementary Information:**

The online version contains supplementary material available at 10.1186/s13195-023-01163-3.

## Background

Alzheimer’s disease (AD) dementia is the most common type of dementia worldwide, accounting for an estimated 60–80% of cases [1]. It has been established that AD may start to develop decades before the onset of clinical symptoms associated with AD dementia [2]. Patients with AD dementia often show a progressive decline in cognitive function, the symptoms of which include memory loss, language difficulties, executive and visuospatial dysfunction, loss of higher-level planning, and intellectual coordination skills [3–5]. Psychological and behavioral symptoms include depression, hallucinations, delusion, and agitation, while instrumental symptoms comprise difficulties with daily activities [4].

AD, the underlying pathology causing AD dementia, is characterized by abnormalities in glutamatergic pathways related to *N*-methyl-d-aspartate (NMDA) receptor dysfunction [6] in the cortical and hippocampal regions of the brain, though NMDA dysfunction is not exclusive to AD [7]. The NMDA receptor plays a pivotal role in the synaptic function underlying learning and memory, and the aforementioned abnormalities have been associated with cognitive impairment [8, 9]. NMDA receptors are activated by binding of both glutamate and glycine at the extracellular ligand binding domain [9, 10]. The inhibition of a pre-synaptic glycine transporter-1 (GlyT1), which functions to regulate synaptic glycine levels, may therefore improve NMDA receptor hypofunction by elevating the levels of extracellular glycine in the synaptic cleft [8, 10]. Increased NMDA receptor signaling results in an increase in long-term potentiation and synaptic plasticity in the hippocampus, amygdala, and medial septum, which may improve cognitive function and memory [11].

Acetylcholinesterase inhibitors (AChEIs) are efficacious symptomatic treatments for mild-to-moderate AD dementia [12] with a modest effect on cognition [12, 13] For moderate-to-severe symptoms, memantine (which functions as both an NMDA receptor antagonist and a dopamine agonist) is often prescribed [5]. However, the symptomatic improvement offered by either AChEIs or memantine is limited [12, 14]. Given the psychosocial impact that declining cognitive function has on patients with AD, there is an unmet need for more effective symptomatic treatments [5].

BI 425809 is a novel potent and selective GlyT1 inhibitor [15, 16]. In animal models, systemic administration of BI 425809 increased glycine levels in rat cerebrospinal fluid, demonstrating functional target engagement, and its use in cognitive tests has shown memory enhancement [15, 16].

This phase II proof-of-clinical concept (PoCC) and dose-ranging study was performed to test the efficacy and safety of a range of doses of BI 425809 in patients with mild-to-moderate probable AD dementia.

## Methods

### Study design

**Fig. 1:**
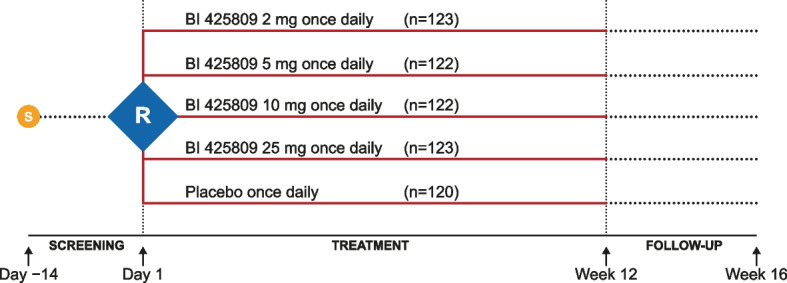
Study design. R, randomized; S, screened population

This was a phase II, 12-week, multicenter, multinational, randomized, double-blind, placebo-controlled, parallel-group comparison in patients with mild-to-moderate probable AD dementia between August 18, 2016, and October 11, 2019 (NCT02788513; EudraCT Number: 2015-005438-24; Fig. 1). Patients were randomized at 97 sites in 14 countries (Austria, Canada, Finland, France, Germany, Greece, Hungary, Italy, Japan, Norway, Poland, Spain, UK, and the USA).

The trial was carried out in compliance with the approved clinical trial protocol, which was in accordance with the principles of the Declaration of Helsinki [17], the International Conference on Harmonisation of Technical Requirements for Registration of Pharmaceuticals for Human Use (ICH) Good Clinical Practice (GCP) guidelines, applicable regulatory requirements, and Boehringer Ingelheim standard operating procedures. All participants provided informed written consent in accordance with ICH GCP and local legislation. All patients had to be able to give informed consent personally and have the capacity for such consent. Each patient also had to have a trial partner who was required to consent separately. The study protocol was reviewed and approved by the local independent ethics committees and relevant local authorities.

#### Patients

This study recruited male or female patients at least 55 years of age with a diagnosis of mild-to-moderate probable AD dementia according to the recommendations from the National Institute on Aging and the Alzheimer’s Association (NIA-AA) workgroups on diagnostic guidelines for AD dementia [18]. Patients were also required to have a Mini-Mental State Examination (MMSE) score of 15–26 at screening. Concomitant use of AChEIs was permitted but not required; those who were currently taking AChEIs were eligible on the condition that they had been using a stable dose for at least 3 months prior to screening, and no change was foreseen for the duration of the study.

Patients who were not currently taking AChEIs but had taken them in the past were also eligible if AChEIs were stopped at least 3 months prior to screening. Patients were required to have a reliable study partner who was in close contact with the patient, available on call, and able to contribute to the Neuropsychological and Clinical Rating Scales at specific study visits. Patients were excluded from participation in the trial if they had dementia secondary to disorders other than AD. Additional exclusion criteria included a hemoglobin level of < 120 g/L (12 g/dL) in men or 115 g/L (11.5 g/dL) in women at screening; those with a history of hemoglobinopathy, such as thalassemia major or sickle cell anemia; those who had taken memantine within 3 months prior to screening; any suicidal behavior in the past 2 years; and suicidal ideation of type 4 or 5 as assessed by the Columbia-Suicide Severity Rating Scale (C-SSRS) in the past 3 months. The full exclusion criteria for this study are listed in Table 1.

**Table 1:** Exclusion criteria

Exclusion criteria	
✗ Dementia secondary to disorders other than Alzheimer’s disease dementia. ✗ Any central nervous system disease other than AD that, according to the investigator, could be associated with worsening cognition. Patients with epileptic seizures in the last 2 years had to be excluded. ✗ A disease or condition which in the opinion of the investigator was likely to interfere with trial testing procedures or put the patient at risk when participating in this trial. ✗ Any documented active or suspected malignancy or history of malignancy with the need of concomitant treatment that interfered with the investigational product. ✗ Patients with a life expectancy of less than 2 years were also excluded. ✗ Any other clinical condition that, in the opinion of the investigator, would jeopardize patient safety while participating in this clinical trial. ✗ Severe renal impairment defined as a GFR < 30 mL/min/1.73 m^2^ at screening. ✗ Hemoglobin less than 120 g/L (12 g/dL) in men or 115 g/L (11.5 g/dL) in women in the screening lab report. History of hemoglobinopathy such as thalassemia major or sickle cell anemia. ✗ Clinically significant uncompensated hearing loss in the judgment of the investigator (use of hearing aids was allowed). ✗ Any suicidal behavior in the past 2 years (i.e., actual attempt, interrupted attempt, aborted attempt, or preparatory acts or behavior). ✗ Any suicidal ideation of type 4 or 5 in the C-SSRS in the past 3 months (i.e., active suicidal thought with intent but without a specific plan or active suicidal thought with plan and intent). ✗ Known history of HIV infection. ✗ Significant history of drug dependence or abuse (including alcohol, as defined in the DSM-V or in the opinion of the investigator) within the last 2 years. ✗ Previous participation in investigational drug studies of dementia of Alzheimer’s type within 3 months prior to screening. Patients having received any active treatment in studies targeting disease modification of AD were excluded. Previous participation in studies with non-prescription medications, vitamins, other nutritional formulations, or non-pharmacological treatments was allowed. ✗ Treatment with restricted medication prior to visit 1 and/or during the screening period. ✗ Planned elective surgery requiring general anesthesia or hospitalization for more than 1 day (requiring an overnight stay) during the study period. ✗ Indication of liver disease, defined by serum levels of either ALT (SGPT), AST (SGOT), or alkaline phosphatase above 3× upper limit of normal as determined during screening.	

*AD* Alzheimer’s disease, *ALT* alanine aminotransferase, *AST* aspartate aminotransferase, *C-SSRS* Columbia-Suicide Severity Rating Scale, *DSM-V* Diagnostic and Statistical Manual of Mental Disorders, Fifth Edition; *GFR* glomerular filtration rate, *HIV* human immunodeficiency virus, *SGOT* serum glutamic oxaloacetic transaminase, *SGPT* serum glutamic pyruvic transaminase

Blood samples obtained from all eligible patients were used for genotyping, including screening for the presence of apolipoprotein E e4 allele (*APOE4*).

#### Randomization

Eligible patients were randomized (1:1:1:1:1) via interactive response technology to one of five groups: BI 425809 2 mg, 5 mg, 10 mg, and 25 mg or placebo once daily (QD) in a 12-week double-blind treatment period (Fig. 1, Fig. S1). Patients were then followed up for an additional 4 weeks, with safety formally evaluated at each visit until the end of the observational period, which was 28 days after the end of treatment or for an appropriately longer time in case of unresolved adverse events (AEs) (Fig. 1). Patients, investigators, and all those involved in trial conduct or analysis, or with any other interest in this double-blind trial, remained blinded to the treatment until after database lock; the randomization code was kept confidential by clinical trial support until this time.

#### Treatments

The dose range was selected based on previous animal cognition tests [16] and a phase I clinical study designed to evaluate the pharmacokinetics and pharmacodynamics of BI 425809 where a dose of BI 425809 10 mg QD produced a mean 50% glycine increase [15]. This corresponded to a target clinical dose of BI 425809 5–10 mg QD.

The trial medication, BI 425809, was manufactured by Boehringer Ingelheim Pharma GmbH & Co KG and provided by a contract research organization. Each patient took three tablets orally QD with water, in the morning, with or without food (Table 2).

**Table 2:** Treatments groups

Group	Treatment regimen	Treatment	Tablets per day
**1**	2 mg BI 425809 QD	1 mg	2-0-0
		25 mg PTM	1-0-0
**2**	5 mg BI 425809 QD	5 mg	1-0-0
		1 and 5 mg PTM	1-0-0
		25 mg PTM	1-0-0
**3**	10 mg BI 425809 QD	5 mg	2-0-0
		25 mg PTM	1-0-0
**4**	25 mg BI 425809 QD	1 and 5 mg PTM	2-0-0
		25 mg	1-0-0
**5**	Placebo QD	1 and 5 mg PTM	2-0-0
		25 mg PTM	1-0-0

*PTM* placebo to match, *QD* once daily

#### Endpoints and assessments

The primary endpoint of efficacy was the change from baseline in Alzheimer’s Disease Assessment Scale-Cognitive Subscale 11 (ADAS-Cog_11_) total score after 12 weeks of treatment. ADAS-Cog_11_ is an 11-item cognitive subscale that objectively assesses memory, language, orientation, and praxis, with a total score range from 0 to 70 (lower scores indicate less severe cognitive impairment) [19]. A negative change indicates an improvement from baseline.

Secondary endpoints included change from baseline in the Alzheimer’s Disease Cooperative Study/Activities of Daily Living (ADCS-ADL) score and the Clinician’s Interview-Based Impression of Change (CIBIC+) score after 12 weeks of treatment. The ADCS-ADL is a 23-item rating scale [20] used to assess basic and instrumental activities of daily living; the overall score can range from 0 to 78, with a lower score indicating greater severity of impairment [21]. The CIBIC+ assesses disease severity and changes and evaluates the behavior, cognition, and function of patients via a semi-structured interview with both the patient and caregiver [22].

A further endpoint was the change from baseline in the Neuropsychiatric Inventory (NPI) score after 12 weeks of treatment. NPI is a neuropsychiatric scale that consists of 10 domains that are rated for both frequency (range: 1–4) and severity (range: 1–3). A composite score for each domain is then calculated (frequency × severity), which ranges from 1–12.

#### Safety

Safety was assessed throughout the study based on the occurrence of AEs (including drug-related AEs, serious AEs [SAEs], and AEs of special interest), vital signs, electrocardiogram and standard laboratory tests, physical examination, neurological examination, and C-SSRS questionnaires.

#### Statistical analyses

Based on one-sided *α* = 0.05, a sample size of 95 evaluable patients per group was needed to identify a standardized effect size of 0.35 with 80% power using a multiple comparison procedure and modeling (MCPMod) approaches. It was planned to add an additional 10% of Japanese patients bringing the total to 105 evaluable patients. Assuming a 10% withdrawal rate, a minimum sample of 117 evaluable patients per group was required. An assumed standardized effect size of 0.35 was used.

The treated set was defined as all patients treated with at least one dose of the trial medication; these patients’ data were analyzed based on the treatment received at randomization. The full analysis set (FAS) was defined as all of the randomized patients who were treated with at least one dose of the trial medication and had a baseline and at least one corresponding post-baseline on-treatment assessment for any efficacy endpoint. These patients were analyzed based on intent-to-treat (i.e., planned treatment assigned at randomization).

An MCPMod approach, in combination with a mixed model of repeated measures (MMRM), was performed for the primary analysis of the primary endpoint. This approach involved the simultaneous comparison of several plausible dose–response models, to evaluate improvements in cognition and to select the best-fitting model(s) for the dose–response relationship for ADAS-Cog_11_ total score over the selected dose range, while protecting the overall probability of type I error. The model included fixed, categorical factors of planned treatment, analysis visit, baseline MMSE stratification factor (≥ 20, < 20), and planned treatment by analysis visit interaction, as well as the continuous fixed covariate of the baseline value, and baseline value by analysis visit interaction. The null hypothesis for the primary endpoint was a flat dose–response pattern across placebo and any dose of BI 425809 within the tested dose range (0–25 mg) for the mean change from baseline to week 12 in ADAS-Cog_11_ total score.

Six pre-defined models were tested: betaMod, Emax, sigEmax, linear, linear in log, and logistic. Except for the betaMod, the maximum effect was assumed to be achieved at the maximum dose tested. If the null hypothesis was rejected, the best-fitting model(s) was refitted to the data without assumptions to generate new estimates of the model parameters. The best-fitting model was identified based on the Akaike Information Criterion. PoCC was established if at least one model was significant.

The secondary analysis of change from baseline in ADAS-Cog_11_ total score at week 12 (primary endpoint) used a restricted maximum likelihood estimation based on MMRM for pairwise comparisons between the treatment groups. This analysis was considered exploratory in nature and was based on the numerical comparison of the respective adjusted treatment means.

Analysis of covariance (ANCOVA) based on observed cases and the last observation carried forward was used for the sensitivity analysis of the primary endpoint. The model included the baseline value for the primary endpoint measure, MMSE stratification factor (≥ 20, < 20) at baseline, and treatment. Similar ANCOVA models based on observed cases were also used for the primary analysis of the secondary endpoints (ADCS-ADL and CIBIC+).

Safety outcomes were analyzed descriptively.

Statistical analyses were conducted using SAS version 9.4 (SAS Institute Inc., Cary, NC, USA).

## Results

### Study population and patient disposition

**Fig. 2:**
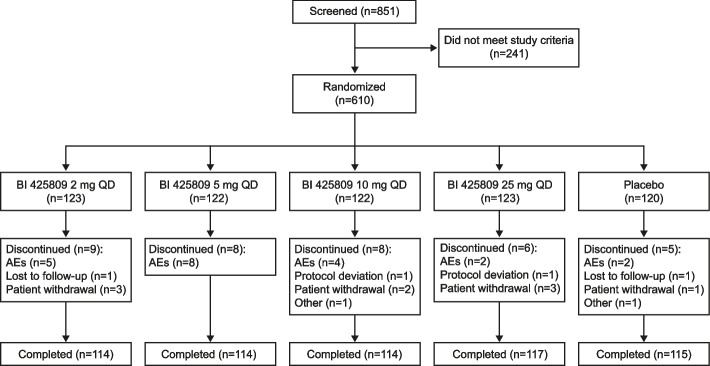
Patient disposition. AE, adverse event; QD, once daily

Of the 851 patients initially screened, 610 patients were randomized into each treatment group: BI 425809 2 mg (*n* = 123), 5 mg (*n* = 122), 10 mg (*n* = 122), and 25 mg (*n* = 123) and placebo (*n* = 120) (Fig. 2). Of the treated patients, 94.1% completed treatment without premature discontinuation and 96.4% completed the trial. Premature discontinuations from trial medication were most frequently due to AEs (3.4%) or because the patient withdrew consent (1.5%).

Treatment compliance was above 97% for all dose groups. In total, 40 (6.6%) patients had at least one important protocol deviation (IPD). The IPDs with an overall frequency > 1% were prohibited medication use during the conduct of the trial (2.3%), participation in an AD trial less than 3 months before screening or with treatment for disease modification (1.1%), and non-compliance with trial medication (1.1%).

Patient demographic data were generally balanced across the treatment groups (Table 3). The mean patient age was 72.9 years (standard deviation [SD], 7.7), 53.1% of the patients were female, patients were predominantly white (81.1 %), the mean time since the first onset of symptoms was 2.3 years (SD, 2.5), and 72.0% and 28.0% of patients had mild or moderate probable AD dementia, respectively. Overall, 49.7% of patients carried *APOE4*.

**Table 3:** Patient demographics

***N*** (%)	BI 2 mg QD (***n*** = 123)	BI 5 mg QD (***n*** = 122)	BI 10 mg QD (***n*** = 122)	BI 25 mg QD (***n*** = 123)	Placebo (***n*** = 120)	Total (***n*** = 610)
**Male**	55 (44.7)	60 (49.2)	56 (45.9)	59 (48.0)	56 (46.7)	286 (46.9)
**Mean (SD) age, years**	72.3 (7.5)	72.5 (8.2)	74.4 (6.9)	72.9 (7.7)	72.4 (7.9)	72.9 (7.7)
**Race**						
**American Indian or Alaska Native**	0 (0.0)	0 (0.0)	0 (0.0)	0 (0.0)	0 (0.0)	0 (0.0)
**Asian**	11 (8.9)	10 (8.2)	12 (9.8)	14 (11.4)	11 (9.2)	58 (9.5)
**Black or African American**	10 (8.1)	5 (4.1)	4 (3.3)	3 (2.4)	8 (6.7)	30 (4.9)
**Native Hawaiian or Pacific Islander**	0 (0.0)	2 (1.6)	1 (0.8)	1 (0.8)	2 (1.7)	6 (1.0)
**White**	97 (78.9)	103 (84.4)	100 (82.0)	102 (82.9)	93 (77.5)	495 (81.1)
**Multiple**	0 (0.0)	0 (0.0)	0 (0.0)	0 (0.0)	0 (0.0)	0 (0.0)
**Missing**	5 (4.1)	2 (1.6)	5 (4.1)	3 (2.4)	6 (5.0)	21 (3.4)
**Mean (SD) body mass index, kg/m**^**2**^	26.7 (4.8)	26.8 (5.5)	26.3 (4.3)	26.0 (4.5)	26.7 (5.6)	26.5 (4.9)
**Disease severity**						
**Mild**	88 (71.5)	88 (72.1)	88 (72.1)	88 (71.5)	87 (72.5)	439 (72.0)
**Moderate**	35 (28.5)	34 (27.9)	34 (27.9)	35 (28.5)	33 (27.5)	171 (28.0)
**MMSE category on the median score**						
**< 22**	58 (47.2)	56 (45.9)	55 (45.1)	60 (48.8)	58 (48.3)	287 (47.0)
**≥ 22**	65 (52.8)	66 (54.1)	67 (54.9)	63 (51.2)	61 (50.8)	322 (52.8)
**Missing**	0 (0.0)	0 (0.0)	0 (0.0)	0 (0.0)	1 (0.8)	1 (0.2)
**Baseline cholinesterase inhibitor use**						
**Yes**	69 (56.1)	78 (63.9)	77 (63.1)	76 (61.8)	81 (67.5)	381 (62.5)
***APOE4*positive,*N*(%)**	64 (52.0)	48 (39.3)	77 (63.1)	62 (50.4)	52 (43.3)	303 (49.7)

^a^Median MMSE score in this population was 22; therefore, the MMSE cutoff was measured at < 22 or ≥ 22

*APOE4* apolipoprotein E4, *MMSE* Mini-Mental State Examination, *QD* once daily, *SD* standard deviation

Baseline data for ADAS-Cog_11_ and MMSE total scores are shown in Table 4.

**Table 4:** Baseline cognitive assessment data

	BI 2 mg QD (***n*** = 123)	BI 5 mg QD (***n*** = 122)	BI 10 mg QD (***n*** = 122)	BI 25 mg QD (***n*** = 123)	Placebo (***n*** = 120)	Total (***n*** = 610)
**ADAS-Cog**_**11**_**total score**						
***N***	123	122	122	123	119	609
**Mean (SD)**	18.8 (7.9)	18.8 (7.4)	19.6 (7.8)	19.6 (7.3)	18.2 (8.0)	19.0 (7.7)
**MMSE total score**						
***N***	123	122	122	123	119	609
**Mean (SD)**	21.3 (3.1)	21.4 (3.1)	21.6 (3.1)	21.5 (3.2)	21.4 (3.0)	21.4 (3.1)

*ADAS-Cog*_*11*_ Alzheimer’s Disease Assessment Scale-Cognitive Subscale 11, *MMSE* Mini-Mental State Exam, *QD* once daily, *SD* standard deviation

### Change from baseline in ADAS-Cog_11_ total score after 12 weeks of treatment

**Fig. 3:**
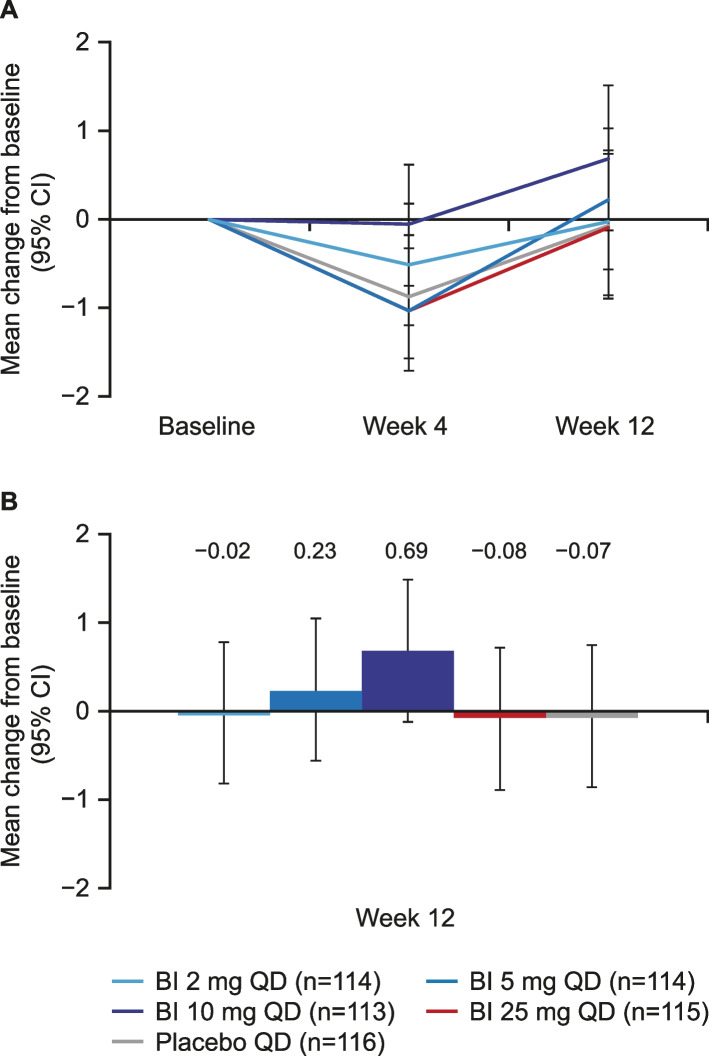
Change from baseline* in ADAS-Cog_11_ total score at both week 4 and week 12 (**A**) and at week 12 only (**B**): MMRM treatment comparison. *Decreases from baseline indicate improvements in ADAS-Cog_11_. ADAS-Cog_11_, Alzheimer’s Disease Assessment Scale-Cognitive Subscale 11; CI, confidence interval; MMRM, mixed model repeated measures; QD, once daily

Of the six dose–response curves evaluated in the MCPMod analysis, none was statistically significant for the primary endpoint (adjusted *p*-value > 0.76 for all models; linear *p* = 0.76; logistic *p* = 0.82; Emax *p* = 0.92; sigEmax *p* = 0.93; linear log *p* = 0.93; betaMod *p* = 0.99; Table 5). There was no dose–response relationship observed across the tested BI 425809 dose range (0–25mg) and placebo for the mean change from baseline to week 12 in ADAS-Cog_11_ total score (Table 5, Fig. 3). Similarly, no significant change from baseline to week 12 in the adjusted mean (MMRM outcomes only) ADAS-Cog_11_ scores was observed (between − 0.08 and 0.69; standard error 0.41) (Fig. 3). The sensitivity analyses using ANCOVA was based on the FAS with last observation carried forward, and observed cases were found to be consistent with the primary analysis.

**Table 5:** Change from baseline in ADAS-Cog_11_ total score: MCPmod test for non-flat dose-response curve

Multiple contrast test^a^	Linear	Logistic	Emax	sigEmax	Linear in log	betaMod
t-Stat	0.0501	− 0.1024	− 0.4898	− 0.5233	− 0.5504	− 1.2587
Adjusted p-value	0.7646	0.8199	0.9225	0.9287	0.9335	0.9931

Data analyzed were the mean change from baseline to week 12 in ADAS-Cog11 total score

*ADAS-Cog*_*11*_ Alzheimer’s Disease Assessment Scale-Cognitive Subscale 11, *MCPMod* multiple comparison procedure and modeling

^a^Critical value for t-Stat: 2.084, alpha = 0.05, one-sided

### Change from baseline in ADCS-ADL and CIBIC+ total score after 12 weeks of treatment

Analysis of adjusted mean change from baseline in ADCS-ADL total score at week 12 found no significant improvement for any tested dose of BI 425809 versus placebo (Table 6). At week 12, patients in the BI 425809 10 mg and 25 mg groups had numerically lower mean scores than at baseline (mean change from baseline for 10 mg: − 1.26; for 25 mg: − 1.90). As the magnitude of the numerical change was small and there was no consistent effect for the other endpoints, this was not interpreted as a clinically meaningful difference. There was no significant improvement in adjusted mean CIBIC+ scores at any dose of BI 425809 compared with the placebo at week 12 (Table 6).

**Table 6:** ADCS-ADL total scores and CIBIC+ scores at week 12: ANCOVA treatment comparison

	**N**	**Adjusted mean change from baseline**	**SE**	**95% CI**		**Adjusted mean difference vs placebo**	**SE**	**95% CI**	
				**Lower**	**Upper**			**Lower**	**Upper**
**ADCS-ADL**^a^									
**BI 425809 2 mg QD**	113	0.29	0.54	− 0.77	1.34	0.02	0.76	− 1.48	1.52
**BI 425809 5 mg QD**	111	0.76	0.54	− 0.31	1.82	0.49	0.77	− 1.01	2.00
**BI 425809 10 mg QD**	110	− 1.26	0.54	− 2.33	− 0.19	− 1.53	0.77	− 3.04	− 0.02
**BI 425809 25 mg QD**	116	− 1.90	0.53	− 2.94	− 0.86	− 2.16	0.76	− 3.65	− 0.67
**Placebo QD**	111	0.27	0.54	− 0.80	1.33	.	.	.	.
**CIBIC+**^b^	**N**	**Adjusted mean**	**SE**	**95% CI**		**Adjusted mean difference vs placebo**	**SE**	**95% CI**	
				**Lower**	**Upper**			**Lower**	**Upper**
**BI 425809 2 mg QD**	114	4.05	0.08	3.90	4.20	− 0.10	0.11	− 0.32	0.11
**BI 425809 5 mg QD**	112	4.10	0.08	3.95	4.25	− 0.05	0.11	− 0.26	0.16
**BI 425809 10 mg QD**	110	4.23	0.08	4.08	4.38	0.08	0.11	− 0.13	0.30
**BI 425809 25 mg QD**	116	4.26	0.08	4.10	4.41	0.10	0.11	− 0.11	0.32
**Placebo QD**	112	4.15	0.08	4.00	4.30	.	.	.	.

*ADCS-ADL* Alzheimer’s Disease Cooperative Study/Activities of Daily Living, *ANCOVA* analysis of covariance, *CI* confidence interval, *CIBIC+* Clinician’s Interview-Based Impression of Change, *CIBIS* Clinical Interview-Based Impression of Severity, *MMSE* Mini*-*Mental State Examination, *QD* once daily, *SE* standard error

^a^The dependent variable was the change from the baseline score at week 12 in ADCS-ADL total score. The model includes fixed, categorical factors of planned treatment and baseline MMSE stratification factor (≥ 20, < 20), as well as fixed continuous covariate of baseline ADCS-ADL score

^b^The dependent variable was the CIBIC+ score at week 12. The model includes fixed, categorical factors of planned treatment and baseline MMSE stratification factor (≥ 20, < 20), as well as fixed continuous covariate of CIBIS score

### Further endpoints

The mean NPI score change from baseline at week 12 was between − 0.90 and 1.33. As the magnitude of change was small, these changes were interpreted as not clinically meaningful (Table 7, Additional file 2: Table S1).

**Table 7:** NPI scores by visit

	BI 425809				Placebo
	2 mg QD	5 mg QD	10 mg QD	25 mg QD	
**Baseline, mean (SD)**	8.09 (9.99)	7.36 (9.03)	8.50 (10.01)	8.03 (10.91)	8.07 (8.11)
**Week 12, mean (SD)**	7.85 (11.97)	6.28 (10.12)	9.34 (11.59)	7.85 (10.42)	7.40 (8.70)
**Change from baseline at week 12, mean (SD)**	− 0.53 (8.20)	− 0.84 (9.49)	1.33 (7.74)	− 0.10 (7.93)	− 0.90 (7.03)

*NPI* neuropsychiatric inventory score, *SD* standard deviation, *QD* once daily

### Safety

BI 425809 was generally well tolerated. Overall, 47.9% (*n* = 292) of patients reported at least one AE during the trial; the frequency of patients with investigator-defined drug-related AEs was similar in all treatment groups, ranging from 15.4 to 19.5% across the BI 425809 treatment groups and 15.8% for placebo (Table 8).

**Table 8:** Summary of AEs

***n*** (%)	BI 2 mg QD (***n*** = 123)	BI 5 mg QD (***n*** = 122)	BI 10 mg QD (***n*** = 122)	BI 25 mg QD (***n*** = 123)	Placebo QD (***n*** = 120)	Total (***n*** = 610)
**Patients with any AE**	57 (46.3)	64 (52.5)	55 (45.1)	62 (50.4)	54 (45.0)	292 (47.9)
**Patients with severe AEs**	3 (2.4)	3 (2.5)	3 (2.5)	2 (1.6)	4 (3.3)	15 (2.5)
**Patients with investigator-defined drug-related AEs**	19 (15.4)	20 (16.4)	22 (18.0)	24 (19.5)	19 (15.8)	104 (17.0)
**Patients with AEs leading to discontinuation of the trial drug**	5 (4.1)	7 (5.7)	4 (3.3)	2 (1.6)	2 (1.7)	20 (3.3)
**Patients with AEs of special interest**	0 (0.0)	0 (0.0)	0 (0.0)	0 (0.0)	1 (0.8)	1 (0.2)
**Patients with serious AEs**	5 (4.1)	4 (3.3)	4 (3.3)	4 (3.3)	5 (4.2)	22 (3.6)
**Fatal**	0 (0.0)	0 (0.0)	0 (0.0)	0 (0.0)	0 (0.0)	0 (0.0)
**Immediately life-threatening**	0 (0.0)	1 (0.8)	0 (0.0)	0 (0.0)	0 (0.0)	1 (0.2)
**Disability/incapacity**	0 (0.0)	0 (0.0)	0 (0.0)	0 (0.0)	0 (0.0)	0 (0.0)
**Required or prolonged hospitalization**	5 (4.1)	3 (2.5)	3 (2.5)	4 (3.3)	1 (0.8)	16 (2.6)
**Congenital anomaly or birth defect**	0 (0.0)	0 (0.0)	0 (0.0)	0 (0.0)	0 (0.0)	0 (0.0)
**Other medically important serious events**	0 (0.0)	1 (0.8)	1 (0.8)	0 (0.0)	4 (3.3)	6 (1.0)
**Patients with other significant AEs (according to ICH E3)**	3 (2.4)	6 (4.9)	3 (2.5)	2 (1.6)	1 (0.8)	15 (2.5)
**Suicidal intent based on C-SSRS**	*n = 123*^a^	*n = 122*^a^	*n = 122*^a^	*n = 122*^a^	*n = 118*^a^	*n = 607*^a^
**Suicidal ideation (1–3)**	3 (2.4)	1 (0.8)	2 (1.6)	2 (1.6)	3 (2.5)	11 (1.8)
**Active suicidal ideation with intent (4–5)**	0 (0.0)	0 (0.0)	0 (0.0)	0 (0.0)	0 (0.0)	0 (0.0)
**Suicidal behavior (6–10)**	0 (0.0)	0 (0.0)	0 (0.0)	0 (0.0)	0 (0.0)	0 (0.0)
**Self-injurious behavior without suicidal intent**	0 (0.0)	1 (0.8)	2 (1.6)	0 (0.0)	1 (0.8)	4 (0.7)
**Eye disorders**	3 (2.4)	0 (0.0)	5 (4.1)	4 (3.3)	3 (2.5)	15 (2.5)
**Hemoglobin decreased**	0 (0.0)	1 (0.8)	1 (0.8)	6 (4.9)	0 (0.0)	8 (1.3)

*AE* adverse event, *C-SSRS* Columbia-Suicide Severity Rating Scale, *ICH* International Conference on Harmonization, *QD* once daily

^a^Number of patients with a post-baseline C-SSRS

In total, 3.6% (*n* = 22) of patients reported at least one SAE, with similar frequencies across the BI 425809 treatment groups and placebo. The only SAE reported for ≥ 0.5% of patients overall was ‘fall’ (4 patients, 0.7%). The frequency of patients with AEs leading to discontinuation of trial medication was low (3.3%), and there were no fatal AEs. AEs by system order class (SOC) and preferred term (PT) were generally balanced across the treatment groups. At the SOC level, the only events with an overall frequency ≥ 5% were ‘nervous system disorders’ (12.8%), ‘infections and infestations’ (12.3%), ‘gastrointestinal disorders’ (11.0%), ‘investigations’ (7.7%), and ‘psychiatric disorders’ (6.9%). At the PT level, AEs with an overall frequency ≥ 2% were headache (5.4%), diarrhea (3.9%), dizziness (3.9%), nasopharyngitis (3.1%), nausea (3.1%), urinary tract infection (2.8%), and fall (2.1%). Eye disorders were limited and observed in 2.5% (*n* = 15) of patients (Table 8). Hemoglobin reduction was observed infrequently between 0.0 and 4.9% in BI 425809-treated patients (Table 8). The maximum effect was a relative change from baseline of 5.7% observed in the BI 425809 25 mg treatment group. A total of 8 patients reported this hemoglobin reduction, 6 of whom were in the BI 425809 25 mg treatment group.

C-SSRS assessments identified no reports of active suicidal ideation (0 patients with C-SSRS scores of 4–5) or suicidal behavior (0 patients with C-SSRS scores of 6–10) during the study. Four patients displayed self-injurious behavior without suicidal intent (Table 8).

## Discussion

The present trial did not demonstrate PoCC for the efficacy of BI 425809 in improving memory, cognitive function, and activities of daily living in patients with probable AD dementia, and therefore, a suitable dose could not be defined in this patient population.

No significant, non-flat dose–response relationships for any of the models used was detected for the change from baseline to week 12 in the ADAS-Cog_11_. Analysis of the secondary endpoints, ADCS-ADL and CIBIC+ scores, did not detect any significant improvement for the BI 425809 treatment groups compared with the placebo. The further endpoint, NPI, also showed no significant improvements versus placebo from baseline to week 12.

BI 425809 was generally well-tolerated with no new safety issues identified. No meaningful differences between the treatment groups or dose dependencies were observed. Transient visual disturbances and central nervous system side effects have previously been noted for GlyT1 inhibitors [23, 24]. However, again, within the SOC ‘eye disorders’, no meaningful differences between the treatment groups were observed, and the frequency of ‘visual impairment’ and ‘dyschromatopsia’ was consistent with previous work on BI 425809 [24]. A decrease in hemoglobin is also a potential risk for BI 425809 according to preclinical data and class effect. GlyT1 is expressed in human erythroid cells where it facilitates heme biosynthesis by transporting extracellular glycine into the cell; thus, inhibition of GlyT1 has an effect on the production of hemoglobin [25]. A dose-dependent decrease in hemoglobin levels was observed. The maximum effect was a decrease of 5.7% observed in the 25 mg BI 425809 treatment group (placebo: slight decrease of 0.5%). However, this did not raise any new safety concerns. Reported suicidal ideation C-SSRS scores remained low (without intent to act).

There are a few potential limitations to this study. The trial was of a relatively short duration (12 weeks); it may be that this period was not long enough to reveal any statistically or clinically relevant effects of treatment. Nonetheless, in the case of a symptomatic effect, one would expect an early response; therefore, a longer trial may not necessarily have yielded different results. Additionally, patients with a broad range of disease severity (mild-to-moderate with MMSE scores ranging from 15 to 26 at screening) were recruited, and the presence or absence of background AChEIs might also impact our findings. However, findings from the numerical analysis of the mild versus moderate probable AD dementia subgroups, as well as the subgroup of patients with concomitant AChEI use, were similar to those of the overall analyses.

Another potential limitation may be that some of the patients recruited for the trial were too advanced in the progression of their disease symptomatology for GlyT1 inhibition to have an effect [26]. It is recognized that for chronic conditions, symptomatic treatment must be administered as early as possible to have a significant effect on symptomatology [26], and this might be particularly pertinent for the patients with the more advanced probable AD dementia in this study.

In 2018, the NIA-AA changed their diagnostic criteria of AD for research purposes from a clinical to biological definition, with biomarker and neuropathological findings now forming the basis of these criteria [2]. Biomarker testing to diagnose the patients via the updated NIA-AA criteria was not conducted at the entry to the present study, and this may pose another limitation to our findings. The lack of positive biomarker evidence of AD can be cited as a concern in dementia studies, as patients who meet the clinical criteria may not in fact have the disease [27]. With growing evidence to suggest that AD is heterogenous, a single approach to treatment might not be the most efficacious line of investigation; identification of subtypes of the disease may guide potential future targets and multifactorial intervention strategies [28, 29]. *APOE4* is a possible therapeutic target, as carriers of this gene are more likely to develop AD [28]. In the present study, patients were tested for *APOE4* (Table 1), with 49.7% of the study population being positive. Previous reports indicate that approximately 40–80% of patients with AD carry at least one copy of *APOE4* [28, 30], suggesting that these patients may be at the lower end of representation in our study, compared with naturalistic samples or other clinical trials. However, results from the *APOE4* population in this study were not notably different from the overall study findings.

## Conclusions

In conclusion, no clinically meaningful changes from baseline in neuropsychological assessments were observed across a range of BI 425809 doses administered to patients with mild-to-moderate probable AD dementia. All treatments were generally well tolerated, and no new safety concerns were identified in the trial.

## Supplementary Information

**Additional file 1: Figure S1.** Patient flow MMSE stratification factor (≥20 vs <20) and MMSE category based on median score (≥22 vs <22).

**Additional file 2: Table S1.** NPI: Descriptive statistics for the 12 individual behavioral domains of NPI score by visit*.

## Data Availability

To ensure independent interpretation of the clinical study results, Boehringer Ingelheim grants all external authors access to relevant material, including participant-level clinical study data, as needed by them to fulfill their role and obligations as authors under the ICMJE criteria. Clinical study documents and participant clinical study data are available to be shared on request after the publication of the primary manuscript in a peer-reviewed journal and if regulatory activities are complete and other criteria met as per the Boehringer Ingelheim Policy on Transparency and Publication of Clinical Study Data (see MyStudyWindow). Bona fide, qualified scientific and medical researchers are eligible to request access to the clinical study data with corresponding documentation describing the structure and content of the datasets. Upon approval, and governed by a legal agreement, data are shared in a secured data access system for a limited period of 1 year, which may be extended upon request. Prior to providing access, clinical study documents and data will be examined and, if necessary, redacted and de-identified, to protect the personal data of study participants and personnel and to respect the boundaries of the informed consent of the study participants. Researchers should use the https://vivli.org/ link to request access to study data and visit Medical & Clinical Trials | Clinical Research| MyStudyWindow for further information.

## Abbreviations

AD: Alzheimer’s disease
NMDA: *N*-methyl-d-aspartate
GlyT1: Glycine transporter-1
AChEIs: Acetylcholinesterase inhibitors
PoCC: Proof-of-clinical concept
ICH: International Conference on Harmonization of Technical Requirements for Registration of Pharmaceuticals for Human Use
GCP: Good Clinical Practice guidelines
NIA-AA: National Institute on Aging and the Alzheimer’s Association
MMSE: Mini-Mental State Examination
C-SSRS: Columbia-Suicide Severity Rating Scale
*APOE4*: Apolipoprotein E e4 allele
QD: Once daily
AE: Adverse event
ADAS-Cog_11_: Alzheimer’s Disease Assessment Scale-Cognitive Subscale 11
ADCS-ADL: Alzheimer’s Disease Cooperative Study/Activities of Daily Living
CIBIC+: Clinician’s Interview-Based Impression of Change
NPI: Neuropsychiatric Inventory
SAE: Serious adverse event
MCPMod: Multiple comparison procedure and modeling
FAS: Full analysis set
MMRM: Mixed model of repeated measures
ANCOVA: Analysis of covariance
IPD: Important protocol deviation
SD: Standard deviation
SOC: System order class
PT: Preferred term
