# Supplementary material for: Efficacy and safety of the novel GlyT1 inhibitor BI 425809 in Alzheimer’s dementia: a randomized controlled trial
Source: Alzheimers Res Ther. 2023 Jan 28;15:24. doi: 10.1186/s13195-023-01163-3 (PMC9883916; doi:10.1186/s13195-023-01163-3)
Supplement: Supplementary file 1 — Additional file 1: Figure S1. Patient flow MMSE stratification factor (≥20 vs <20) and MMSE category based on median score (≥22 vs <22). [file 13195_2023_1163_MOESM1_ESM.docx]

**Supplementary Figure 1:** Patient flow MMSE stratification factor (≥20 vs <20) and MMSE category based on median score (≥22 vs <22)


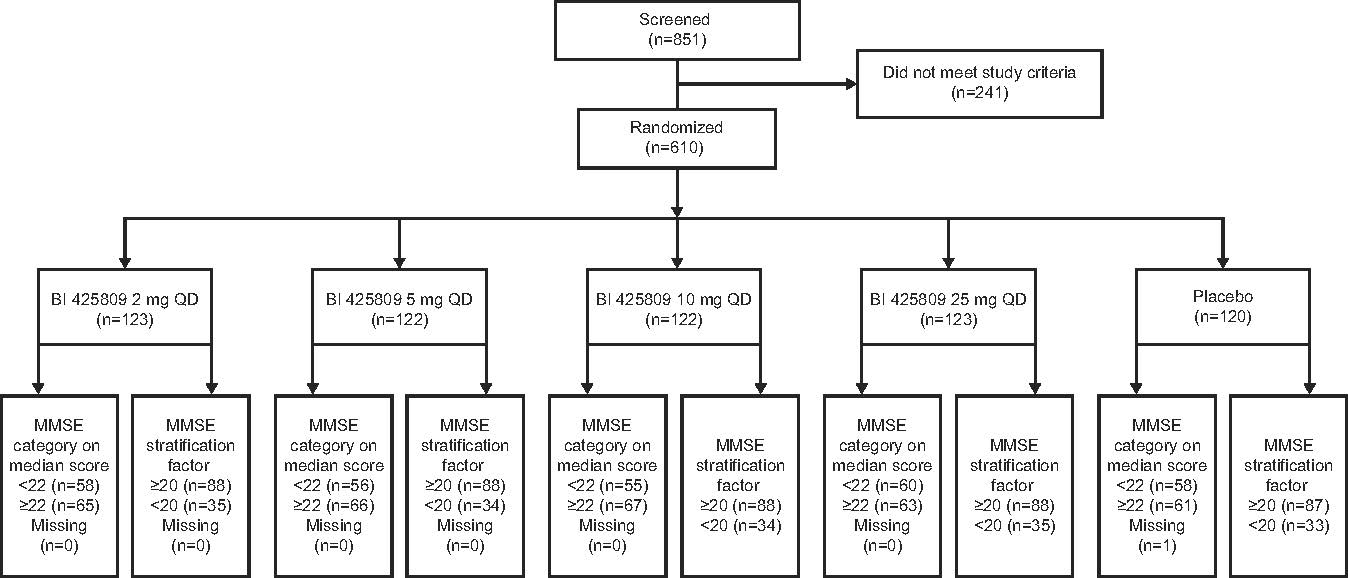
MMSE, mini-mental state examination.
