# Supplementary material for: Efficacy and safety of the novel GlyT1 inhibitor BI 425809 in Alzheimer’s dementia: a randomized controlled trial
Source: Alzheimers Res Ther. 2023 Jan 28;15:24. doi: 10.1186/s13195-023-01163-3 (PMC9883916; doi:10.1186/s13195-023-01163-3)
Supplement: Supplementary file 2 — Additional file 2: Table S1. NPI: Descriptive statistics for the 12 individual behavioral domains of NPI score by visit*. [file 13195_2023_1163_MOESM2_ESM.docx]

**Supplementary Table 1:** NPI: Descriptive statistics for the 12 individual behavioral domains of NPI score by visit*

|  | **BI 425809** | | | |  |
| --- | --- | --- | --- | --- | --- |
|  | **2 mg QD** | **5 mg QD** | **10 mg QD** | **25 mg QD** | **Placebo** |
| **Aberrant Motor Behavior FxS Score** |  |  |  |  |  |
| **Baseline, mean (SD)** | 0.51 (1.7) | 0.59 (1.93) | 0.59 (1.8) | 0.94 (2.37) | 0.55 (1.68) |
| **Week 12, mean (SD)** | 0.73 (2.14) | 0.52 (1.87) | 0.80 (1.92) | 0.84 (2.02) | 0.79 (1.97) |
| **Agitation/Aggression FxS Score** |  |  |  |  |  |
| **Baseline, mean (SD)** | 0.97 (1.95) | 0.33 (0.97) | 0.69 (1.45) | 0.74 (1.64) | 0.77 (1.59) |
| **Week 12, mean (SD)** | 0.63 (1.59) | 0.27 (0.88) | 0.69 (1.51) | 0.66 (1.43) | 0.51 (1.12) |
| **Anxiety FxS Score** |  |  |  |  |  |
| **Baseline, mean (SD)** | 0.73 (1.66) | 0.63 (1.78) | 0.86 (1.89) | 0.73 (1.78) | 0.84 (1.56) |
| **Week 12, mean (SD)** | 0.68 (1.71) | 0.57 (1.92) | 0.69 (1.42) | 0.57 (1.47) | 0.84 (1.86) |
| **Apathy/Indifference FxS Score** |  |  |  |  |  |
| **Baseline, mean (SD)** | 1.50 (2.31) | 1.50 (2.91) | 1.82 (2.78) | 1.72 (2.69) | 1.27 (2.13) |
| **Week 12, mean (SD)** | 1.53 (2.76) | 1.22 (2.62) | 2.17 (3.32) | 1.68 (2.92) | 1.27 (2.31) |
| **Appetite/Eating Disorders FxS Score** |  |  |  |  |  |
| **Baseline, mean (SD)** | 0.98 (2.39) | 1.28 (2.77) | 1.31 (2.77) | 0.77 (2.18) | 1.16 (2.63) |
| **Week 12, mean (SD)** | 0.72 (1.87) | 0.82 (2.27) | 1.34 (2.73) | 0.85 (2.16) | 1.05 (2.40) |
| **Delusions FxS Score** |  |  |  |  |  |
| **Baseline, mean (SD)** | 0.41 (1.75) | 0.47 (1.88) | 0.35 (1.56) | 0.29 (1.27) | 0.25 (1.01) |
| **Week 12, mean (SD)** | 0.58 (2.02) | 0.47 (1.72) | 0.28 (1.37) | 0.36 (1.61) | 0.19 (0.89) |
| **Depression/Dysphoria FxS Score** |  |  |  |  |  |
| **Baseline, mean (SD)** | 0.96 (1.76) | 0.83 (1.66) | 0.95 (1.68) | 1.06 (2.17) | 1.05 (1.57) |
| **Week 12, mean (SD)** | 0.81 (1.74) | 0.92 (2.29) | 1.24 (2.30) | 0.98 (2.03) | 0.96 (1.65) |
| **Disinhibition FxS Score** |  |  |  |  |  |
| **Baseline, mean (SD)** | 0.22 (1.02) | 0.32 (0.96) | 0.20 (0.90) | 0.32 (1.15) | 0.25 (0.83) |
| **Week 12, mean (SD)** | 0.31 (1.42) | 0.16 (0.94) | 0.23 (0.71) | 0.35 (1.42) | 0.37 (1.27) |
| **Elation/Euphoria FxS Score** |  |  |  |  |  |
| **Baseline, mean (SD)** | 0.11 (0.51) | 0.13 (0.82) | 0.17 (0.88) | 0.10 (0.56) | 0.19 (0.90) |
| **Week 12, mean (SD)** | 0.12 (0.68) | 0.05 (0.42) | 0.10 (0.49) | 0.10 (0.76) | 0.12 (0.61) |
| **Hallucinations FxS Score** |  |  |  |  |  |
| **Baseline, mean (SD)** | 0.07 (0.40) | 0.20 (1.03) | 0.21 (1.25) | 0.07 (0.43) | 0.06 (0.56) |
| **Week 12, mean (SD)** | 0.12 (0.69) | 0.27 (1.18) | 0.19 (0.78) | 0.10 (0.48) | 0.03 (0.29) |
| **Irritability/Lability FxS Score** |  |  |  |  |  |
| **Baseline, mean (SD)** | 0.92 (1.89) | 0.55 (1.41) | 0.76 (1.65) | 0.71 (1.73) | 0.87 (1.95) |
| **Week 12, mean (SD)** | 1.03 (2.21) | 0.49 (1.33) | 0.75 (1.61) | 0.85 (2.12) | 0.78 (1.45) |
| **Sleep/Night Behavior Disorder FxS Score** |  |  |  |  |  |
| **Baseline, mean (SD)** | 0.72 (1.85) | 0.54 (1.34) | 0.60 (1.55) | 0.60 (1.69) | 0.81 (2.16) |
| **Week 12, mean (SD)** | 0.62 (1.75) | 0.54 (1.66) | 0.86 (2.24) | 0.51 (1.85) | 0.51 (1.75) |

*Based on cohort numbers as follows: Baseline: 2 mg QD (n=121), 5 mg QD (n=120), 10 mg QD (n=121), 25 mg QD (n=120), placebo (n=118); Week 12: 2 mg QD (n=113), 5 mg QD (n=112), 10 mg QD (n=110), 25 mg QD (n=116), placebo (n=111).

FxSs, function; SD, standard deviation.
